# Apical ballooning and cardiomyopathy in a melanoma patient treated with ipilimumab: a case of takotsubo-like syndrome

**DOI:** 10.1186/s40425-015-0048-2

**Published:** 2015-02-17

**Authors:** Benjamin P Geisler, Roy A Raad, Diana Esaian, Elad Sharon, David R Schwartz

**Affiliations:** New York University School of Medicine, New York, NY 10016 USA; National Cancer Institute, Bethesda, 20892 MD USA

**Keywords:** Takotsubo cardiomyopathy, Ipilimumab [Supplementary concept], Melanoma, Drug-related side effects and adverse reactions

## Abstract

Although animal studies have shown that the immunomodulator ipilimumab causes inflammation of the myocardium, clinically significant myocarditis has been observed only infrequently. We report a case of suspected acute coronary syndrome without a culprit lesion on cardiac angiography and takotsubo cardiomyopathy (TC)-like appearance on echocardiography in a patient with metastatic melanoma who received four standard doses of ipilimumab. Apical ballooning, hyperdynamic basal wall motion, systolic anterior motion of the mitral valve, and associated severe left ventricular outflow tract obstruction were present. Restaging with positron emission tomography-computed tomography done soon after discharge incidentally revealed increased fludeoxyglucose uptake in the apex. This case illustrates that a TC-like syndrome might be caused by autoimmune myocarditis after ipilimumab treatment although this was not biopsy-confirmed. Post-marketing surveillance should capture cardiac events occurring in patients treated with ipilimumab to better document and clarify a relationship to the drug, and biopsies should be considered. Physicians utilizing this novel agent should be aware of the potential for immune-related adverse events.

## Background

Derived from Japanese word for octopus pot, typical takotsubo cardiomyopathy (TC) presents clinically indistinguishable from acute coronary syndrome, but systolic apical ballooning of a hypo- or akinetic left ventricular (LV) apex with hyperdynamic basal walls will be present from the deleterious effects of a catecholamine surge. In 90% of cases, a clear emotional or physical stressor precedes the presentation, hence the term stress-induced cardiomyopathy [1]. Acute increased adrenergic activity from cocaine, pheochromocytoma, sub-arachnoid hemorrhage, or trauma can precipitate TC via altered vascular tone and/or direct toxicity. Patients experience typical chest pain and may manifest heart failure or shock. Troponin release, often small, and anterior ST segment elevations are usually present. Angiography, per definition, should fail to reveal a culprit lesion. In 16% of cases there is a pressure gradient across a narrowed LV outflow tract, often associated with systolic anterior motion of the mitral valve (SAM). We report a case of “takotsubo cardiomyopathy-like” myocardial dysfunction after ipilimumab treatment for metastatic malignant melanoma.

## Case presentation

**Figure 1:**
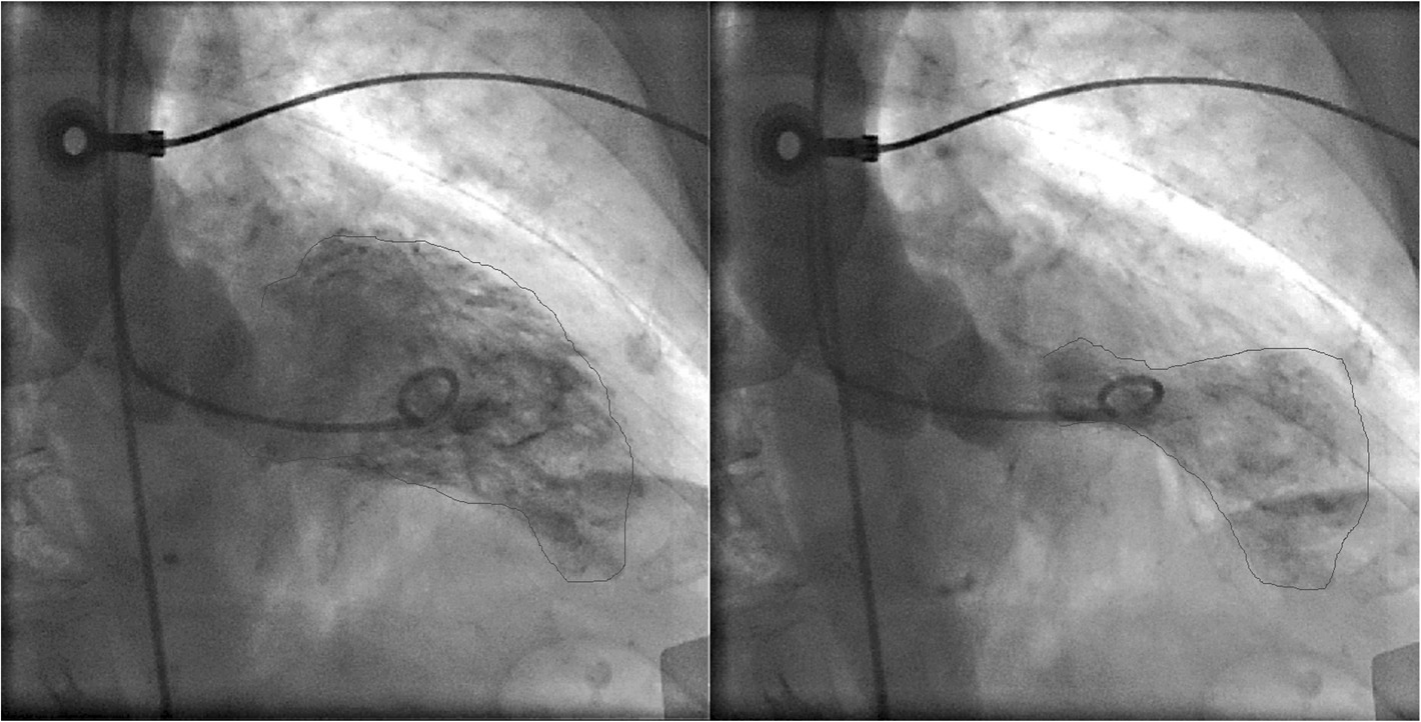
**Ventriculogram during diastole (left) and systole (right).** While the left ventricular apex in these two images appears nearly akinetic, the remaining left ventricle is hyperkinetic.

**Figure 2:**
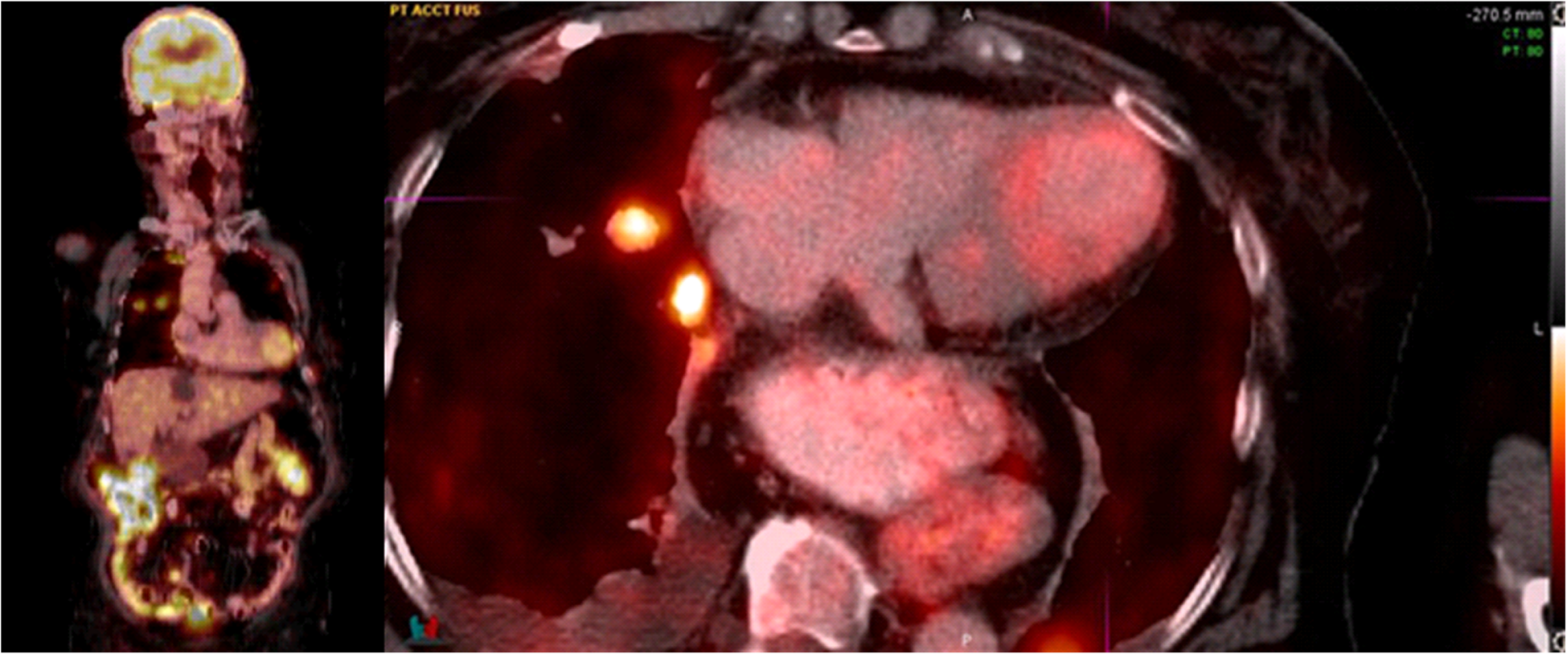
**Coronal (left) and axial (right) PET/CT showing a focus of mildly increased FDG uptake corresponding to ballooning of the left ventricular apex.** Additional findings at the axial level through the chest include FDG-avid metastatic lung nodules in the right upper lobe, small right pleural effusion and a large hiatal hernia.

An 83-year old woman with hypertension was diagnosed with biopsy-proven vaginal melanoma four months prior to admission. PET-CT showed invasive loco-regional disease and a three-millimeter nodule in the left upper lung lobe. Attempted resection was complicated by positive margins. Four cycles of ipilimumab (3 mg/kg every three weeks), last dosed three weeks prior to hospitalization, were administered. Radiotherapy was deferred. The patient had developed pruritus, lethargy, and malaise after the third dose and diarrhea after the fourth dose of ipilimumab. These symptoms responded to short courses of prednisone. Prior to admission, the patient experienced two weeks of fairly continuous worsening substernal chest pain and progressive dyspnea. On admission, she denied acute emotional stress, illicit drug or herbal medication use. Electrocardiography revealed sinus tachycardia at 110/minute and 1 millimeter ST elevations in leads I, V_2_, and V_3_. The initial troponin-I level was 0.98 (normal <0.04) ng/ml, thyroid-stimulating hormone was measured as 2.6 (0.4-4.0) mIU/L, and the erythrocyte sedimentation rate was 65 (<20) mm/hour. A chest radiograph revealed numerous round bilateral lung masses. Transthoracic echocardiography showed an akinetic apex, hyperkinetic base and septum, an ejection fraction of 50%, and LV outflow tract obstruction with a peak gradient of 100 mmHg with SAM. Emergent cardiac angiography demonstrated an isolated 30% proximal left anterior descending artery stenosis without evidence of a thrombus. No intervention was performed. Figure 1 displays the ventriculogram. The patient developed transient supraventricular and ventricular tachycardia. A beta-blocker and was started. On hospital day 3, the patient was asymptomatic and was transferred to acute cardiac rehabilitation. ^18^F-Fludeoxyglucose (FDG) PET-CT performed two days later revealed focal FDG uptake in the patient’s ballooned LV apex (Figure 2).

## Discussion and conclusion

Drug-induced TC has been associated with direct-acting sympathomimetic xenobiotics, causing myocardial dysfunction either directly, due to free radical formation and apoptosis, or via alterations in coronary vasomotion; atropine and adrenergic reuptake inhibitors may do the same. Chemotherapeutics and monoclonal antibodies potentially implicated have included 5-fluorouracil, rituximab, and vascular endothelial growth factor antagonists. Postulated mechanisms include direct myocardial ischemia due to coronary vasospasm, toxic myocarditis from impurities, upregulation of transforming growth factors stimulating myocytal reticulin fiber growth, and increased inflammatory cytokine levels [2,3].

Ipilimumab, a monoclonal antibody directed against cytotoxic T-lymphocyte-associated antigen 4 (CTLA-4), leads to activated T-lymphocyte proliferation and results in prolonged overall survival in metastatic melanoma [4]. Almost three quarters of patients on ipilimumab experience immune-related adverse events, most commonly rash, pruritus, diarrhea, and colitis. One third of these are severe, life-threatening, or disabling [5]. CTLA-4-deficient mice succumb to myocarditis and pancreatitis characterized by lymphoproliferative infiltrates and granular tissue formation [6,7]. Clinically significant myocarditis has been identified in less than one percent of patients [8]. Although the proposed Mayo Clinic criteria for TC precludes myocarditis [9], a TC-like appearance on echocardiography has been reported in lymphocytic myocarditis without evidence of a viral cause [10], suggesting that TC is a nosographic entity with more than one etiology.

In the absence of a known stressor, considering the subacute onset of symptoms, and supportive imaging, we hypothesize that autoimmune myocarditis from ipilimumab-related CTLA-4 inactivation could have accounted for our patient’s presentation of a “takotsubo cardiomyopathy-like” syndrome. Though the PET-CT was for restaging and not protocolled as a cardiac study, focal FDG uptake in the LV apex, corresponding to the akinetic myocardium, is dramatic. Focal uptake of FDG, representing enhanced metabolic activity, may be due to microvascular or myocyte damage, changes in fatty acid utilization, or a combination [11]. As expected, most non-inflammatory TC cases in the literature assessed with cardiac PET-CT show decreased FDG uptake in the stunned areas [12,13]. However, a recently reported case linked increased FDG uptake with severely decreased fatty acid metabolism in an impaired, inflamed myocardium [14]. When done early, a positive PET-CT has a 100% specificity compared to endomyocardial biopsy for acute myocarditis [15]. However, since no biopsy was taken, a cardiac metastasis cannot be excluded although most cases are clinically silent and cardiovascular manifestations are rarely seen in isolation or as a presenting symptom; and cardiac lesions are usually multiple at diagnosis [16].

Medline and Embase searches as well as a query to the manufacturer of ipilimumab failed to reveal similar cases. Therefore, to our knowledge, this might be the first reported case of a “takotsubo cardiomyopathy-like” syndrome in a patient treated with ipilimumab. While no causal relationship can be proven, post-marketing surveillance should capture cases of ipilimumab cardiac toxicity and physicians utilizing this novel agent should be aware of this potential immune-related adverse event.

## Consent

Written informed consent was obtained from the patient for publication of this case report and any accompanying images. A copy of the written consent is available for review by the Editor-in-Chief of this journal.

## Abbreviations

CTLA-4: Cytotoxic T-lymphocyte-associated antigen 4
FDG: ^18^F-Fluorodeoxyglucose
LV: Left ventricular
PET-CT: Positron emission and computed tomography
SAM: Systolic anterior motion of the mitral valve
TC: Takotsubo cardiomyopathy
